# Clinical tools for evaluating congenital adrenal hyperplasia in resource-limited hospitals: a study at a tertiary hospital in Saudi Arabia

**DOI:** 10.3389/fendo.2026.1730774

**Published:** 2026-02-06

**Authors:** Daniah Alhazmi, Azzam Alabdulqader, Shahad Almeqbel, Raghad Alhuthil, Afaf Alsagheir

**Affiliations:** 1 Section of Pediatric Endocrine/Metabolism, Department of Pediatrics, King Faisal Specialist Hospital and Research Center, Riyadh, Saudi Arabia; 2 Department of Pediatrics, King Faisal Specialist Hospital and Research Center, Riyadh, Saudi Arabia; 3 College of Medicine, Alfaisal University, Riyadh, Saudi Arabia

**Keywords:** 17-OHP, androstenedione, bone age, clinical control, limited resources

## Abstract

**Background:**

Congenital adrenal hyperplasia (CAH) treatment is complicated by hormonal imbalances, necessitating a dual therapeutic approach to both correct cortisol deficiency and manage androgen overproduction. Unfortunately, hospitals with limited resources lack some necessary standard laboratory tests to manage patients with CAH.

**Objective:**

To investigate the interrelation between different monitoring strategies in clinical practice for managing patients with CAH.

**Methods:**

This prospective cross-sectional study involved children with CAH caused by 21-hydroxylase deficiency (21-OHD) treated at King Faisal Specialist Hospital and Research Centre. KFSHRC is not resource-limited; the proposed recommendations are intended for settings that lack full biochemical panels. Univariable, bivariable, and multivariable logistic regression were done for association testing.

**Results:**

The cohort included 96 children with 21-OHD, predominantly female (61.5%), with a median age of 6 years. Adrenal crises occurred in 20.8% of patients. Most participants were treated with hydrocortisone (97.9%) and fludrocortisone (88.5%), with high reported treatment compliance (90.6%). Biochemical abnormalities were observed in 26% for ACTH, 21.9% for 17-OHP, and 17.7% for testosterone. Biochemical control was significantly associated with treatment compliance (OR 7.6, p = 0.03). In adjusted analyses, androstenedione, ACTH, and testosterone control were strongly associated with 17-OHP control (all p < 0.01). Regarding skeletal outcomes, older age was inversely associated with bone age control, whereas clinical control (OR 11.1, p < 0.01) and controlled androstenedione levels (OR 3.0, p = 0.04) were independent predictors of optimal bone age.

**Conclusion:**

Based on these findings, we recommend integrating growth velocity monitoring and androstenedione testing into routine visits as valuable indicators for assessing clinical control in 21-OHD children. Yet, larger studies are needed to validate simplified monitoring frameworks for resource-limited hospitals.

## 1 Introduction

Congenital adrenal hyperplasia (CAH), which is caused by 21-hydroxylase deficiency (21-OHD), is among the most common adrenal disorders (1). This autosomal recessive disease results in cortisol and aldosterone deficiency and excess androgen production (1). CAH manifests as either severe CAH, typically known as classic CAH, or mild/late-onset CAH, also known as nonclassic CAH. Classic CAH can be subclassified into salt-wasting (SW) or simple virilizing subtypes based on the extent of aldosterone deficiency (2, 3). In addition to 21-OHD, CAH encompasses a spectrum of rarer enzymatic defects, including deficiencies of 11β-hydroxylase, 3β-hydroxysteroid dehydrogenase, 17α-hydroxylase, P450 oxidoreductase, and steroidogenic acute regulatory protein (StAR) (4).

The general characteristics of CAH include adrenal insufficiency, genital ambiguity or disordered sex development, SW crises, infertility, short stature, and an elevated risk of metabolic syndrome during adolescence and adulthood (5). The standard of care for CAH involves physiological glucocorticoid replacement to correct cortisol deficiency and suppress ACTH-driven adrenal androgen excess. In SW forms, mineralocorticoid replacement and sodium supplementation are necessary to maintain electrolyte balance (6).

Globally, CAH is considered a rare genetic disorder with an incidence of approximately 1 in 10,000–20,000 live births (7). In contrast, national newborn screening data from Saudi Arabia reported a higher incidence of 1 in 6,400 births (8). Consequently, CAH represents a relatively common and clinically significant adrenal disorder within the Saudi population, posing ongoing diagnostic and management challenges, as demonstrated by multiple local studies (8–12).

Typically, CAH is usually managed via follow-up visits in which clinical and laboratory parameters are assessed (13). Clinical parameters include history and physical examination. Symptoms of adrenal crises such as salt cravings, irregular menstrual cycles in female patients, fatigue, nausea, and abdominal pain are considered in the diagnosis. Meanwhile weight, height, growth velocity, blood pressure, signs of virilization, tanner stage, and the presence or absence of hyperpigmentation are investigated in the physical examination.

Concerning laboratory parameters, ACTH, androstenedione, testosterone, and 17-hydroxyprogesterone (17-OHP) are evaluated in every visit (13). However, the use of these traditional biochemical markers in CAH monitoring has several important limitations. 17-OHP exhibits marked diurnal variation and is highly dependent on the timing of sample collection relative to glucocorticoid dosing, which complicates interpretation and may lead to misclassification of disease control; furthermore, target ranges are poorly standardized across centers (14). Androstenedione levels correlate with adrenal androgen production but are influenced by age, sex, pubertal status, and assay variability, limiting their reliability as a standalone marker of control. Testosterone is particularly problematic in children and females, as levels may remain within normal ranges despite poor adrenal control and may be confounded by gonadal production, especially during puberty, reducing its specificity for adrenal androgen excess (14). ACTH concentrations show extreme circadian and pulsatile variability and are highly sensitive to recent glucocorticoid intake, rendering single measurements unreliable for routine monitoring and poorly correlated with clinical outcomes. Collectively, these limitations highlight that these biomarkers reflect short-term hormonal fluctuations rather than integrated metabolic control, supporting the need for cautious interpretation and complementary clinical and radiological assessment in CAH management (14).

Therefore, we investigated the interrelationships among different monitoring strategies (clinical, biochemical, and bone maturation), as well as predictors of controlled bone age, in clinical practice for managing patients with CAH. This study will help policymakers develop a standardized clinical approach based on clinical, bone age and laboratory data to help resource-limited hospitals manage patients with CAH to optimize care with fewer and more cost-effective investigations.

## 2 Materials and methods

This prospective snapshot, cross-sectional study involved pediatric patients (0–14 years), including infants, with CAH caused by 21-OHD. CAH patients caused by other enzymatic defects were excluded from analysis. Data were collected prospectively once from medical records and routine clinic visits at the endocrine clinic of King Faisal Specialist Hospital and Research Center (KFSHRC, Riyadh, Saudi Arabia) for patients who presented in the clinic between July 2023 and July 2024.

KFSHRC is a large referral hospital, it is not resource-limited; the proposed recommendations are intended for settings that lack full biochemical panels.

Informed consent and assent were obtained from all participants and their families. Institutional Review Board approval was secured from KFSHRC (reference number 2231108).

The collected data encompassed demographic information, medical history, clinical presentations, growth metrics, physical examinations, laboratory results, and bone age assessments. The CDC growth chart was used as a reference range to evaluate growth metrics (15).

### 2.1 Variable definitions

Clinical control was defined as (1) maintenance of age- and sex-appropriate linear growth velocity, defined as growth within ±1 standard deviation (SD) of Centers for Disease Control and Prevention (CDC) growth velocity reference standards for chronological age and pubertal stage (15); (2) absence of adrenal crises requiring emergency medical intervention during the follow-up period; and (3) absence of persistent electrolyte abnormalities, specifically hyponatremia or hyperkalemia, on routine clinical assessment, and adequate mineralocorticoid replacement.

Bone age is considered within the normal (controlled) range when it is within ±2 standard deviations (SDS) of chronological age (16).

Regarding biochemical control, we used routinely measured morning laboratory values as part of standard CAH follow-up. In our center, patients are instructed to take their morning hydrocortisone dose prior to blood draw, and all samples are obtained in the morning.

Patients were classified as biochemically uncontrolled if any of the following values fell outside acceptable institutional ranges: 17−OHP > 35 nmol/L (applied across all ages), ACTH outside 5–60 ng/L, testosterone above age-/sex-appropriate ranges (with pubertal male levels considered physiologic), or androstenedione above Tanner stage–specific thresholds. Androstenedione thresholds were defined using age-, sex-, and pubertal stage–adjusted reference ranges (17), with prepubertal levels corresponding to Tanner Stage I and increasing ranges thereafter through Tanner Stage V. For males, reference limits spanned from <51 ng/dL (<1.8 nmol/L) in Tanner I (<9.8 years) to 65–210 ng/dL (2.3–7.3 nmol/L) in Tanner V (12.8–17.3 years). For females, limits ranged from <51 ng/dL (<1.8 nmol/L) in Tanner I (<9.2 years) to 80–240 ng/dL (2.8–8.4 nmol/L) in Tanner V (11.8–18.6 years). Tanner Stages II–IV were also captured using published ranges (17). Values above these ranges were considered elevated, indicating increased adrenal androgen activity.

Growth measurements (height and weight) were obtained by trained pediatric nurses during routine visits using standardized stadiometers and calibrated digital scales. Androstenedione was measured in the clinical biochemistry laboratory using a commercially available Elecsys Androstenedione immunoassay kit (Roche Diagnostics). Bone age was assessed using left hand and wrist radiographs and interpreted by a board-certified radiologist according to the Greulich and Pyle standards.

### 2.2 Statistical analysis

Data were securely stored in REDCap (10.8.0 - ^©^ 2021 Vanderbilt University). Statistical analysis was performed using STATA (v.18, StataCorp, College Station, TX, USA). Descriptive statistics were presented as frequencies and percentages, medians and interquartile ranges, and means and standard deviations (SDs), as appropriate. For lab values below the detection level, half of the cut-off was taken. Univariable, bivariable, and multivariable logistic regression were done for association testing. A p-value lower than 0.05 was considered statistically significant.

## 3 Results

The cohort included 96 21-OHD children confirmed by *CYP21A2* genotyping, including 59 girls (61.5%) and 37 boys (38.5%), with a median age of 6 years. Most participants (89%) were diagnosed with SW CAH. The majority of cases were diagnosed at birth (77.1%). A positive family history was reported in 34.4% of patients. Meanwhile, 20.8% of patients experienced adrenal crises, with 85% of these patients having 1–2 crises/year (**Table 1**).

**Table 1:** Demographics and clinical background of the study participants (n = 96).

Characteristics	n (%), median [IQR]
Sex	
- Female	59 (61.5)
- Male	37 (38.5)
Current age (years)	6 [3.5, 8]
Diagnosis	
- SW CAH	86 (89.6)
- Simple virilizing (non-SW) CAH	10 (10.4)
Genetic cause*	
- 21-OHD	96 (100)
Age at diagnosis	
- Prenatal	1 (1.0)
- At birth	74 (77.1)
- 1–3 weeks	21 (21.9)
Positive family history	33 (34.4)
History of adrenal crisis	20 (20.8)
- 1–2 crises	17 (85.0)
- 3–4 crises	3 (15.0)

*21-hydroxylase deficiency confirmed by CYP21A2 genotyping.

Regarding growth data, height SDS was near the population mean (median −0.3 [IQR −1.2 to 0.8]), and mid-parental height SDS was comparable (median −0.6 [IQR −1.0 to 0.01]). Weight SDS showed a mild positive shift (median 0.6 [IQR −0.4 to 1.6]). Among the three patients with final height data, median final height SDS was −1.2 [IQR −1.5 to −0.8], falling within the lower normal range (**Table 2**).

**Table 2:** Growth data and Tanner staging.

Growth and Tanner data	n (%), median [IQR]
Mid-parental height (SDS)	−0.6 [−1.0, 0.01]
Height (cm)	118.6 [97.8, 131]
Height (centile)	37.1 [11.7, 80.5]
Height (SDS)	−0.3 [−1.2, 0.8]
Final height (cm) (n = 3)	151.1 [148.1, 154]
Final height (SDS) (n = 3)	−1.2 [−1.5, −0.8]
Weight (kg)	23.4 [15.4, 37]
Weight (centile)	72.9 [28.7, 94]
Weight (SDS)	0.6 [−0.4, 1.6]
Body mass index	18 [16, 21]
Growth velocity (cm/year)	7.1 [5.1, 8.9]
Pubic hair Tanner stage	
- Stage 1	67 (69.8)
- Stage 2	14 (14.6)
- Stage 3	3 (3.1)
- Stage 4	3 (3.1)
- Stage 5	3 (3.1)
- Not applicable/Not done	6 (6.3)
Breast development Tanner stage (n = 59) ^a^	
- Stage 1	49 (83.1)
- Stage 2	3 (5.1)
- Stage 3	1 (1.7)
- Stage 4	2 (3.4)
- Stage 5	1 (1.7)
- Not applicable/Not done	3 (5.1)
Genitalia Tanner stage	
- Stage 1	73 (76.0)
- Stage 2	4 (4.2)
- Stage 3	3 (3.1)
- Stage 4	1 (1.0)
- Stage 5	2 (2.1)
- Not applicable/Not done	13 (13.5)
Stretched penile length (cm)^b^	4.3 (SD: 1.3)
Clinically uncontrolled	11 (11.5)

a Reported for girls.

b Reported for boys.

Tanner stage 1 pubic hair was observed in 69.8% of participants, whereas genitalia and breast development mostly remained at stage 1 (76% and 83.1%, respectively). The stretch penile length among males averaged 4.3 cm (**Table 2**).

Hydrocortisone was used by 97.9% of participants, with 69.2% receiving thrice-daily doses. Fludrocortisone was administered to 88.5% of patients, while 9.4% of patients received leuprorelin. Compliance with therapy was high (90.6%), although 18.8% of participants had uncontrolled biochemical parameters (**Table 3**).

**Table 3:** Management and biochemical data.

Management and biochemical data	n (%)
17-OHP (nmol/L)	
- Males, median [IQR]	0.7 [0.2, 11.4]
- Females, median [IQR]	10 [0.4, 42]
- No. of uncontrolled	21 (21.9%)
Testosterone (nmol/L)	
- Males, median [IQR]	0.045 [0.045, 0.045] ^a^
- Females, median [IQR]	0.045 [0.045, 0.36] ^b^
- No. of uncontrolled	17 (17.7%)
Androstenedione (nmol/L)	
- Males, median [IQR]	0.5 [0.5, 1] ^b^
- Females, median [IQR]	1.1 [0.5, 6.6]
- No. of uncontrolled	22 (22.9%)
ACTH (ng/L)	
- Males, median [IQR]	14.1 [3, 79.9]
- Females, median [IQR]	15.3 [5.5, 54.9]
- No. of uncontrolled	25 (26.0%)
Overall biochemically uncontrolled	18 (18.8%)^c^
Hydrocortisone usage (n = 94)	94 (97.9%)
Frequency	
Once daily	2 (2.1%)
Twice daily	27 (28.7%)
Thrice daily	65 (69.2%)
Hydrocortisone total daily dose (mg/m^2^/day), mean ± SD	13.7 ± 4.2
Body surface area (m^2^), mean ± SD	0.86 ± 0.35
Prednisone	2 (2.1%)^d^
Fludrocortisone usage	85 (88.5%)
- Dose (mg), mean ± SD	0.11 ± 0.03
Letrozole	2 (2.1%)
Other medications	
- Leuprorelin	9 (9.4%)
- NaCl	4 (4.2%)
- Flutamide	1 (1.0%)
- Keppra	1 (1.0%)
- Growth hormone	1 (1.0%)
Compliance with therapy	87 (90.6%)

Data are presented as frequency and percentage [n (%)] for categorical variables, and as median [IQR] or mean ± SD for continuous variables, as appropriate.

17-OHP: 17-Hydroxyprogesterone, ACTH: Adrenocorticotropic Hormone, IQR: Interquartile Range, SD: Standard Deviation, NaCl: Sodium Chloride.

a Uniform low testosterone concentrations in males reflect prepubertal physiology; all male patients were biochemically controlled.

b Identical median and lower IQR values reflect left-skewed distributions with clustering at physiologic low values.

c 17 are females, and one is male. The overall biochemically uncontrolled definition is the same as the biochemically uncontrolled definition stated in the methods.

d Prednisone: Two patients were on prednisone twice daily (e.g., 5 mg AM/2.5 mg PM; 10 mg AM/5 mg PM).

Concerning laboratory data, 26% of patients had elevated ACTH levels, 21.9% had elevated 17-OHP levels, and 17.7% had abnormal testosterone levels (**Table 3**). Univariable logistic regression revealed no significant association between laboratory and clinical control (odds ratio (OR): 0.4, *p* = 0.40). Yet, the biochemical control was associated with treatment compliance (OR: 7.6, p=0.03) (**Table 4**).

**Table 4:** Univariable logistic regression analysis.

Parameters	OR [95% CI]	*p*
Laboratory vs. clinical control	0.4 [0.05, 3.34]	0.40
Laboratory vs. treatment compliance	7.6 [1.17, 49.46]	0.03*

CI: confidence interval.

*Statistical significance.

**Table 5** presents the results of bivariate logistic regression analysis evaluating biochemical predictors of 17-OHP control adjusted for sex. Androstenedione control displayed a strong association with 17-OHP control (OR = 11.0, *p* < 0.01). ACTH control (OR = 30.6, *p* < 0.01) and testosterone control (OR = 8.1, *p* < 0.01) were also significantly associated with 17-OHP control.

**Table 5:** Bivariable logistic regression analysis of the biochemical predictors of 17-OHP control adjusted for sex.

Parameters	OR [95% CI]	*p*
Androstenedione control	11.0 [3.54, 34.21]	<0.01*
ACTH control	30.6 [7.20, 129.85]	<0.01*
Testosterone control	8.1 [2.35, 27.9]	<0.01*

CI: confidence interval.

*Statistical significance.

Furthermore, when investigating predictors of bone age control, in univariable analysis, older age was associated with lower odds of bone age control (OR: 0.9, p = 0.02), while clinical control (OR 7.5, p = 0.01) and controlled androstenedione levels (OR: 3.1, p = 0.03) were significantly associated with improved bone age control. Female gender, 17-OHP control, ACTH control, and testosterone control were not significant predictors. In the multivariable model, age remained inversely associated with bone age control (OR: 0.9, p = 0.03), while clinical control showed a strong independent association (OR 11.1, p < 0.01), and controlled androstenedione remained a significant predictor (OR 3.0, p = 0.04) (**Table 6**).

**Table 6:** Logistic regression analysis: predictors of bone age control.

Predictor	Univariable analysis			Multivariable analysis		
	OR	95% CI	p-value	OR	95% CI	p-value
Female gender	1.2	0.54–2.84	0.61	–	–	–
Age (years)	0.9	0.76–0.97	0.02*	0.9	0.75–0.98	0.03*
Clinical control	7.5	1.51–36.69	0.01*	11.1	2.13–57.38	<0.01*
Androstenedione controlled	3.1	1.13–8.19	0.03*	3.0	1.04–8.84	0.04*
17-OHP controlled	1.0	0.38–2.68	0.99	–	–	–
ACTH controlled	0.7	0.27–1.76	0.43	–	–	–
Testosterone controlled	3.0	1.00–8.94	0.05	–	–	–

OR: odds ratio, CI: confidence interval.

*Statistical significance.

## 4 Discussion

This study provided valuable insights into the clinical characteristics, management outcomes, and biochemical control of pediatric patients with CAH caused by 21-OHD. A key focus was identifying the most effective predictors of both biochemical stability and bone age control.

The observed lack of an association between laboratory and clinical outcomes Aligns with the results of small series studies (18, 19). An analysis of international data from the International-Congenital Adrenal Hyperplasia/Disorders Of Sex Development registry (n = 345) revealed large variability in 17-OHP and androstenedione levels between different centers and no correlations between these biomarkers and weight SDS (20).

Bone age assessment remains essential for monitoring treatment adequacy in CAH (21). It was selected as a key outcome in this study because it represents a clinically meaningful indicator of cumulative androgen exposure over several months (22, 23) and is widely used in CAH monitoring research (24, 25). However, bone age should not be interpreted in isolation, as linear growth may remain near-normal in children receiving physiologic glucocorticoid replacement despite biochemical variability (26, 27). In our cohort, the median height SDS was −1.2, consistent with near-normal growth. This supports earlier work by Hendricks et al., who emphasized that combining clinical assessment, growth velocity, and skeletal maturation provides a more reliable gauge of glucocorticoid adequacy than biochemical markers alone (27).

We also observed no significant associations of 17-OHP and ACTH levels with bone age, in line with previous research highlighting the unreliability of a single 17-OHP measurement because of its daily fluctuations (28). 17-OHP is a steroid hormone that indicates adrenal gland activity in CAH and reflects ACTH production by the pituitary gland. However, current data suggest that 17-OHP levels do not accurately represent cortisol levels in the bloodstream, making it inappropriate as the sole measure for assessing and adjusting treatment dosages (29).

Interestingly, androstenedione control was positively correlated with 17-OHP control in this study, in line with prior results (20). In addition, Kang et al. (28) explored the relationship between 17-OHP and androstenedione, hormones that respond differently to low-dose ACTH stimulation in patients with 21-OHD. Their study identified a strong correlation between baseline 17-OHP and androstenedione levels independent of sex, deficiency type, or the time of sampling. The strongest association was observed in the morning, at which time point measurements of androstenedione exhibited a strong correlation with 17-OHP (r^2^ = 0.81). The fold change increase in 17-OHP levels after ACTH injection, but not that of androstenedione, was negatively associated with the basal 17-OHP level. They concluded that the random serum 17-OHP level, as applied in the clinic, is a reliable guide, whereas the low-dose ACTH stimulation test has minimal value in monitoring patients with 21-OHD (28).

Furthermore, we found an association between testosterone and 17-OHP levels corrected for sex, as reported by Turcu et al. (30), who found that testosterone was correlated with 17-OHP when adjusted for age and sex, although it had a weaker correlation than androstenedione.

Most patients in our cohort were treated with hydrocortisone administered in three divided doses, consistent with standard pediatric practice (13, 31, 32). Although hydrocortisone remains the preferred glucocorticoid due to its short half-life and reduced risk of growth suppression, dosing schedules and total daily doses varied across patients. This variability, together with the observed association between biochemical control and treatment compliance, further highlights the limitations of isolated hormone measurements and supports the value of integrated clinical indicators that reflect cumulative hormonal exposure over time.

Current CAH guidelines advocate comprehensive monitoring strategies that integrate growth velocity, pubertal staging, bone age assessment, and biochemical testing (33). Growth velocity is emphasized as a key clinical parameter, as it captures both glucocorticoid overtreatment (growth suppression) and undertreatment (accelerated growth and skeletal maturation). While 17-OHP is recognized as a central biochemical marker, existing guidelines provide limited practical guidance regarding the role and monitoring frequency of androstenedione, particularly in resource-limited settings (33).

From a feasibility perspective, our findings support the pragmatic use of growth velocity and androstenedione as complementary indicators of disease control. Growth velocity is universally obtainable and reflects integrated hormonal exposure over time (13), while androstenedione demonstrated consistent associations with both biochemical stability and skeletal outcomes in this cohort. Building on current guideline principles and clinical experience (6, 33), we propose a tiered monitoring strategy for resource-constrained settings: routine assessment of growth velocity (every 3 months during the first 2 years of life and every 6 months thereafter); measurement of androstenedione levels 1–2 hours after the morning dose of hydrocortisone; comprehensive clinical examination at each visit; and periodic bone age assessment every 2 years after age 4, or annually if the patient demonstrates rapid growth or clinical concerns. This approach balances feasibility, cost-effectiveness, and clinical relevance while remaining aligned with established CAH care principles.

Unlike prior studies that describe routine CAH monitoring parameters, this study provides an evidence-based prioritization of monitoring tools by evaluating their relative associations with clinically meaningful outcomes. Rather than proposing new biomarkers, our innovation lies in identifying a simplified, pragmatic monitoring framework suitable for resource-limited settings. We demonstrate that growth velocity and androstenedione, both accessible and interpretable, are independently associated with bone age control, whereas isolated measurements of 17-OHP and ACTH were not. This approach shifts clinical focus from exhaustive biochemical testing toward integrated indicators that better reflect cumulative hormonal exposure and long-term disease control.

However, several limitations warrant consideration. The single-center, cross-sectional design limits causal inference and generalizability. Additionally, emerging biomarkers such as the androstenedione/testosterone ratio and 11-oxygenated androgens were not evaluated, as they were not routinely done during the study period. Therefore, while growth velocity and androstenedione appear valuable for monitoring, they should be interpreted as part of a broader clinical framework rather than standalone indicators.

Future studies should focus on large, multicenter, longitudinal cohorts incorporating serial or profiled biomarker measurements, including salivary or urinary steroids, to better characterize dynamic hormonal exposure and its relationship with long-term outcomes such as final adult height, metabolic health, and bone mineral density. As highlighted by Bacila et al., further evaluation of emerging biomarkers, particularly 11-oxygenated androgens, is essential to refine monitoring strategies in CAH (14).

## 5 Conclusion

In summary, our study emphasized the importance of comprehensive monitoring in CAH management, specifically advocating for a nuanced approach that considers multiple indicators of health rather than relying solely on bone age or hormonal levels alone. Clinical control, as assessed by growth velocity, was significantly associated with bone age. Additionally, androstenedione control was a better predictor of biochemical stability and bone age control in this study, whereas 17-OHP and ACTH levels exhibited no associations with bone age. Thus, this study recommends integrating growth velocity and androstenedione testing into routine visits as valuable indicators for assessing 21-OHD control. This targeted approach enhances clinical management, aligning with patient-centered care in the context of CAH.

## Data Availability

The raw data supporting the conclusions of this article will be made available by the authors, without undue reservation.
